# Biocompatibility and osteointegration capability of β-TCP manufactured by stereolithography 3D printing: *In vitro* study

**DOI:** 10.1515/biol-2022-0530

**Published:** 2023-01-24

**Authors:** Jialiang Li, Jiaxi Li, Yubing Yang, Xijing He, Xinyu Wei, Qinghua Tan, Yiqun Wang, Siyue Xu, Sue Chang, Weiwei Liu

**Affiliations:** Department of Orthopedics, The Second Affiliated Hospital of Xi’an Jiaotong University, Xi’an, Shaanxi Province, China; Department of Health Management, The Second Affiliated Hospital of Xi’an Jiaotong University, Xi’an, Shaanxi Province, China; Department of Precision Medicine Group, Equipment Research Institute, National Innovation Institute of Additive Manufacturing, Xi’an, Shaanxi Province, China

**Keywords:** β-TCP, bioceramics, stereolithography, biocompatibility, osteogenesis

## Abstract

Beta-tricalcium phosphate (β-TCP) bioceramics have an inorganic composition similar to the human bone. While conventional methods can only produce ceramic scaffolds with poor controllability, the advancement of 3D-printing, especially stereolithography, made it possible to manufacture controllable, highly precise, micropore ceramic scaffolds. In this study, the stereolithography was applied to produce β-TCP bioceramics, while ZrO_2_, Al_2_O_3_, Ti6Al4V, and polyetheretherketone (PEEK) were used as controls. Phase analysis, water contact angle tests, and Micro-CT were applied to evaluate the surface properties and scaffold. Hemolytic toxicity, cell proliferation, and morphological assessment were performed to evaluate the biocompatibility. Alkaline phosphatase (ALP) level, mineralization, and qRT-PCR were measured to evaluate the osteointegration. During the manufacturing of β-TCP, no evident impurity substance and hemolytic toxicity was found. Cells on β-TCP had good morphologies, and their proliferation capability was similar to Ti6Al4V, which was higher than the other materials. Cells on β-TCP had higher ALP levels than PEEK. The degree of mineralization was significantly higher on β-TCP. The expression of osteogenesis-related genes on β-TCP was similar to Ti6Al4V and higher than the other materials. In this study, the β-TCP produced by stereolithography had no toxicity, high accuracy, and excellent osteointegration capability, thus resulting as a good choice for bone implants.

## Graphical abstract

**Figure:**
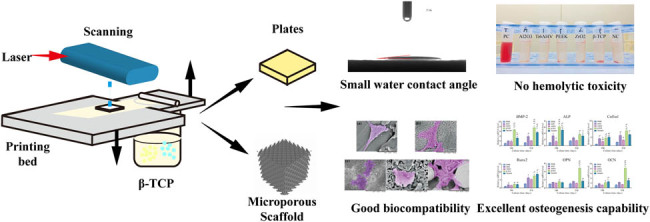

## 1 Introduction

The incidence of bone diseases, such as trauma, tumor, and degeneration, has been gradually increasing every year, resulting in ever-higher requirements for bone substitutes in clinical practice [1,2], and stem cell-based bone regeneration [3,4]. Autogenous bone graft is the gold standard of the bone implant; however, the source of autogenous bone is limited, and harvesting could induce secondary injuries. Therefore, an autogenous bone graft is rarely used to treat large segmental bone defects [5]. Metal bone implants, such as Ti–6Al–4V (Ti6Al4V, a class of medical titanium alloys), could provide stable mechanical support and good clinical effects [1,6,7]. According to Wolff’s Law (the growth, absorption, and reconstruction of bone are related to the mechanical stress of bone), however, metal has a far higher elastic modulus than human bones and thus could lead to stress-shielding, consequently inducing osteoporosis [8–10]. In addition, metal debris could also induce the aseptic loosening of implants [11]. Polymer materials such as polyetheretherketone (PEEK) have a similar elastic modulus as human bones and have been widely applied in spinal, oral, and maxillofacial surgeries [12,13]. Yet, PEEK could promote the formation of fibrous tissue with surrounding tissues, thus influencing the efficiency of osteointegration of bone implants [14,15].

The major inorganic components of human bone are calcium phosphate (CaP), which makes it possible to treat bone defects by bioceramics [16]. Some commonly used bioceramic materials, such as ZrO_2_ and Al_2_O_3_, are characterized by high toughness, corrosion resistance, and wearing resistance and are mainly used in oral and maxillofacial surgeries [17,18]. Hydroxyapatite (HA) is one of the components of human bone that has high biocompatibility and osteogenesis capability. However, HA is difficult to process and has poor degradability, which challenges its use as a bone implant [19,20]. On the other hand, beta-tricalcium phosphate (β-TCP), one of the CaP ceramics, has better osteogenesis capability and degradability than HA, making it to become a good choice for bioceramic material of bone implants [21,22].

Micropore structures are required for bone implants as they provide spaces for the growth of cells and blood vessels and consequently maintain long-term stability [23–25]. Due to the high melting point and toughness, as well as poor flexibility, the sophisticated micropore structures are difficult to be manufactured for bioceramics. The conventional methods, which include gas foaming, particulate leaching, and freeze-drying, can be used to produce ceramic scaffolds with high porosity. Nonetheless, the morphological parameters of micropores are uncontrollable, and the interconnection of micropores cannot be guaranteed, which is unfavorable for the growth of cells and tissues [26–28]. Fortunately, the advancement of 3D printing (3DP) techniques has made it possible to manufacture sophisticated, highly precise micropore structures [29–33]. The layer-by-layer printing also favors maintaining the integrity of micropore structures. In order to improve the mechanical properties, it is necessary to increase the solid components of the ceramic powder-photosensitive resin composite slurry in the printing process. However, this inevitably increases the viscosity of the slurry, consequently reducing the controllability of printing [34].

The application of stereolithography (SLA) technique allows for the rapid whole-surface curing of ceramic slurry and thus ensures the controllability and precision of printing with high-viscosity ceramic slurry. In this study, SLA was applied for the processing of β-TCP material, while ZrO_2_ and Al_2_O_3_ ceramics, Ti6Al4V, and PEEK materials that are commonly used in clinical practices were selected as controls. Phase analysis and surface properties assay, as well as cellular, biochemical, and molecular biological experiments, were performed to evaluate the biocompatibility and osteointegration capability of β-TCP produced by SLA, thus providing evidence for its application in bone implants.

## 2 Materials and methods

### 2.1 Sample preparation and scaffold structure observation

**Figure 1:**
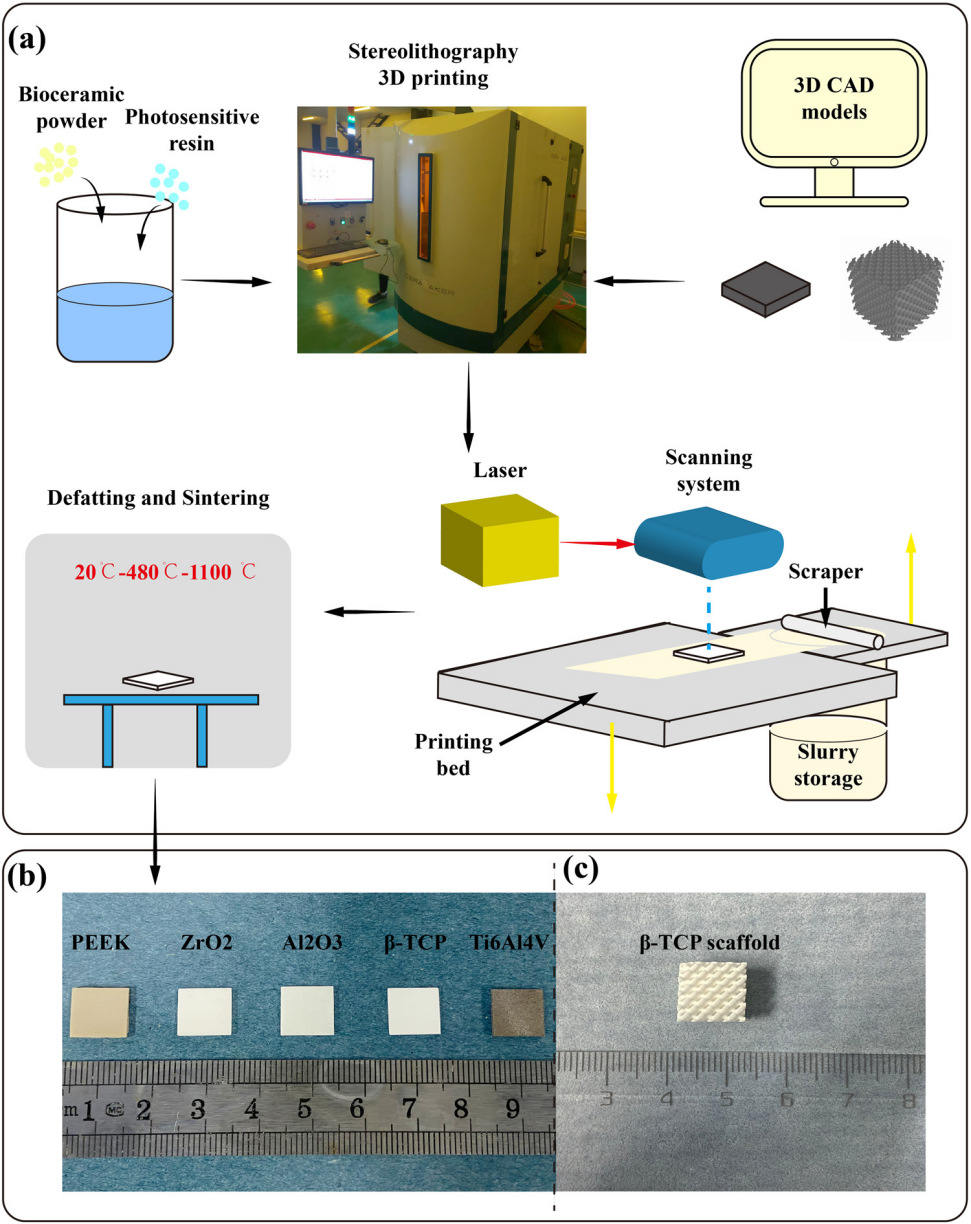
Schematic of the SLA system and example of plates and scaffold used in this experiment. (a) Schematic of SLA 3DP process of β-TCP bioceramics: the composite slurry of β-TCP powder and photosensitive resin was evenly distributed on the printing bed; then laser shooting, curing, and molding were performed according to the CAD models; after that, the 3DP of the next layer continued; finally, the printed ceramic was further processed by defatting and sintering. (b) Thin plates of PEEK, ZrO_2_, Al_2_O_3_, β-TCP, and Ti6Al4V. (c) β-TCP scaffold printed by SLA.

As shown in Figure 1a, the micrometer-scale β-TCP powder and photosensitive resin (National Innovation Institute of Additive Manufacturing, China) were mixed, stirred, ground, and then filtered to acquire the composite slurry (solid content >50%). The CAD models were designed in 3-Matic (Materialise Inc., Leuven, Belgium), and then 3DP was performed by the SLA equipment (Ceramaker, 3D Ceram, France). The printing process was as follows: first, the slurry storage was elevated, and the composite slurry was brought into the printing bed and smoothed by a scraper; then laser shooting, curing, and molding were performed; after that, the printing bed was downshifted to repeat the previous steps; finally, the 3D curing printing layer by layer was completed. The printed ceramic was further processed by defatting and sintering to remove the chemical additives. ZrO_2_ and Al_2_O_3_ ceramics were also manufactured by SLA technique (Xi’an Particle Cloud Biotechnology Co., Ltd, China). Ti6Al4V was printed by selective laser melting technique (National Innovation Institute of Additive Manufacturing, China). PEEK was manufactured by fused deposition modeling (College of Mechanical Engineering, Xi’an Jiaotong University, China). All materials were processed into thin plates for subsequent tests, and β-TCP was processed into a scaffold to preliminarily detect its possibility as a microporous bone implant. The structure of β-TCP scaffold was scanned by Micro-CT (YXLON, Germany) and reconstructed by VGstudio Max 3.0 (Volume Graphics, Germany) software. The scanning parameters were set as follows: X-ray source voltage, 90 kV; beam current, 50 μA; and scanning resolution, 12 μm. Meanwhile, the surface morphology of scaffold was observed using scanning electron microscope (SEM) (JSM-7900F, JEOL, Japan) after gold spraying. The size of the plates was 10 mm for length and width and 1 mm for thickness. All the plates were washed and sterilized before being used in the experiments. Three repeats of plates for each material were used in this study.

### 2.2 X-ray diffraction (XRD)

The β-TCP plates produced by SLA 3DP were carefully washed and dried. Then XRD analysis (Empyrean, PANalytical, Netherlands) was used to scan the β-TCP plates. The scanning range was 4–75°.

### 2.3 Water contact angle measurement

The water contact angle was measured and recorded using a dynamic photography system (JC2000DM, Powereach, China). The contact angles were measured and calculated when the water droplets just contacted (0 s water contact angles) and at 1 s after contacting the surface of a material (1 s water contact angles). Three points were selected for detection on each plate, and three replicates were set for each group.

### 2.4 Hemolytic toxicity

A total of 2% rabbit red blood cell (RBC) suspension was centrifuged and resuspended by an equal volume of PBS as the negative control group or resuspended by distilled water as the positive control group. The different material plates were added to the negative group as the experimental group. The tubes were placed at 37°C for 1 h, after which they were centrifuged, and the optical density (OD) of the supernatant was assessed by a microplate reader (SYNERGY, BioTek, USA) (*n* = 3). The hemolysis rates were calculated as the ratio of the difference between the experimental group and the negative group and the difference between the experimental group and the positive group.

### 2.5 Culture and differentiation of MC3T3 E1 cells

MC3T3-E1 cells were provided by the Procell Life Science & Technology Co., Ltd (Wuhan, China), and cultured in alpha-minimum essential medium (α-MEM) containing 10% fetal bovine serum (Gibco, USA). A total of 1 mL of cell suspension with a density of 10,000 cells/mL medium was added to the surface of the plates in a 24-well culture plate. The cell proliferation capability and morphologies were observed on 1, 3, 5, and 7 days of culture, and the cell differentiation induction began on the seventh day after culture. The cell differentiation-inducing system (Gibco, USA) was the α-MEM, which consisted of 10% fetal bovine serum, 10–8 mol/L dexamethasone, 10 mmol/L β-sodium 3-phosphoglycerate, and 50 mg/L vitamin C. The osteogenesis capability was measured on 7, 14, and 21 days of differentiation induction. All the cells were cultured at 37°C, 5% CO_2_, and the culture medium was changed every other day.

### 2.6 Proliferation and morphologies of MC3T3 E1 cells

Cell Counting Kit-8 (CCK8; KeyGEN BioTECH, China) was used to assess the proliferation capability of the cultured cells (*n* = 3). In brief, 500 µL highly sensitive CCK8 work solution was added to the 24-well culture plate to immerse the plates, which were then incubated at 37°C in dark. After 2 h, the reaction solution was thoroughly mixed and 100 µL was absorbed into a 96-well plate. The OD of each well was measured at 450 nm. The plates with cells were added into 2.5% glutaraldehyde (Solarbio Science & Technology, China) and then placed at 4°C for 4 h. Ethanol solution of 30, 50, 70, 80, 90, and 100% was added sequentially for gradient dehydration. Afterward, the plates were dried under vacuum and gold-sputtered. The morphology of plates and attached cells were visualized by SEM (TM4000, Hitachi, Japan).

### 2.7 Cell differentiation

Cells on plates were digested by trypsin (Solarbio Science & Technology, China) and collected into an Eppendorf centrifugal tube (EP) tube, centrifuged, and then resuspended by double distilled water. The EP tubes were sequentially placed in liquid nitrogen, −20°C, and room temperature, so as to lyse the cells by repeated freezing and thawing. After centrifugation at 4°C, the alkaline phosphatase (ALP) levels were measured by the Alkaline Phosphatase Assay Kit (Beyotime, China) (*n* = 3). The extracellular matrix mineralization was detected by the quantitative method (*n* = 3). In brief, the cells were fixed by 4% paraformaldehyde, after which alizarin red S (Solarbio Science & Technology, China) was added to stain the mineralized nodules. Next, the mineralized nodules were dissolved by 10% hexadecylpyridinium chloride sodium phosphate solution (Solarbio Science & Technology, China), incubated at room temperature for 10 min, and then measured at 562 nm. At the same time, the same quantitative analysis was used for materials without seed cells to eliminate the effect of calcium ion released by the material itself on the results.

### 2.8 qRT-PCR

Osteogenesis-related genes, including bone morphogenetic protein-2 (BMP-2), ALP, Collagen I α1 (COL-1), Runt-related transcription factor 2 (Runx-2), Osteopontin (OPN), and Osteocalcin (OCN), were measured. Cells on plates were collected, and 1 mL TRNzol was added to lyse the cells. The top 400 μL of liquid was collected and an equal volume of chloroform was added, then thoroughly mixed and centrifuged again, and the upper layer was harvested. Isopropanol and 75% ethanol were sequentially added and then dried to acquire the precipitate. Next, RNase-free ddH_2_O was added to dissolve the precipitate, and the concentration and purity of the extracted nucleic acid were measured (Thermo Fisher Scientific, USA). Reverse transcription was performed by using a kit (HiFiScript cDNA Synthesis Kit, Cowin Bio, China), and the primers are shown in Table 1. The amplification system was prepared according to the real-time PCR kit (UltraSYBR Mixture, Cowin Bio, China), and the amplification conditions were as follows: initial denaturation at 95°C for 10 min, denaturation at 95°C for 15 s, and annealing and extension at 60°C for 40 s. Forty cycles of PCR reactions were performed. The relative level of the target gene to the housekeeping gene GAPDH (2^−ΔΔt^) was calculated for statistical analysis (*n* = 3).

**Table 1:** Primer sequences for RT-qPCR

Gene	Forward primer	Reversed primer
BMP-2	5′-AAGCGTCAAGCCAAACACAAACAG-3′	5′-GAGGTGCCACGATCCAGTCATTC-3′
ALP	5′-CTTGGTGGTCACAGCAGTTGGTAG-3′	5′-CCAGGCGACAGGTGAAGAAACAG-3′
OPN	5′-ATGGACGACGATGATGACGATGATG-3′	5′-CTTGTGTACTAGCAGTGACGGTCTC-3′
OCN	5′-CAAGCAGGAGGGCAATAAGGTAGTG-3′	5′-CGGTCTTCAAGCCATACTGGTCTG-3′
COL-1	5′-AACTTTGCTTCCCAGATGTCCTAT-3′	5′-CTCGGTGTCCCTTCATTCCAG-3′
RUNX-2	5′-TCCCGTCACCTCCATCCTCTTTC-3′	5′-GAATACGCATCACAACAGCCACAAG-3′
GAPDH	5′-GGTGAAGGTCGGTGTGAACG-3′	5′-CTCGCTCCTGGAAGATGGTG-3′

### 2.9 Statistical analysis

SPSS 19.0 software (SPSS Inc., Chicago, IL, USA) was used for statistical analysis. Quantitative data were described by mean ± standard deviation. One-way analysis of variance followed by Tukey’s *post hoc* tests was used to analyze the statistically significant differences. *P* < 0.05 was considered statistically significant.

## 3 Results

### 3.1 Characterization of plate samples and porous β-TCP scaffolds

**Figure 2:**
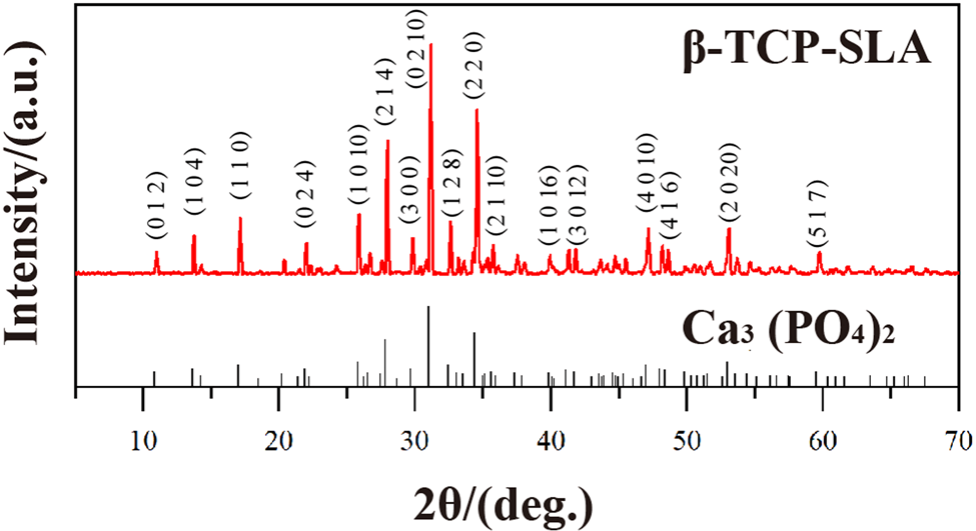
XRD analysis of the β-TCP plates produced by SLA 3DP.

**Figure 3:**
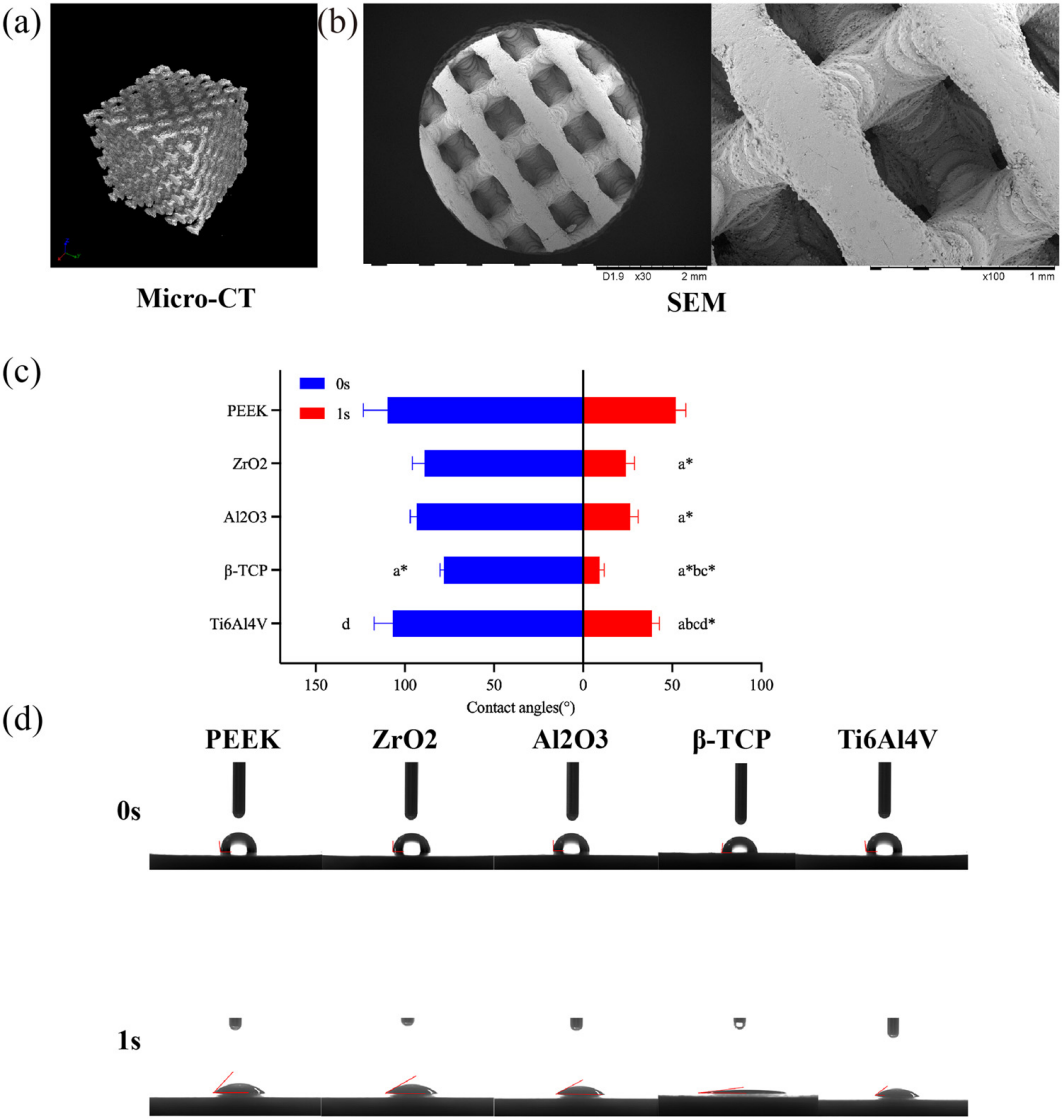
Characteristics of β-TCP scaffold and water contact angle measurement. (a) Micro-CT reconstructed model of β-TCP scaffold. (b) SEM observation of β-TCP scaffold. (c) Comparison of water contact angles of different material plates. (d) Water contact angle recorded and measured 0 and 1 s after contacting the material surface. *P*(*a*) < 0.05 and *P*(*a* ^*^) < 0.01 compared with PEEK; *P*(*b*) < 0.05 compared with ZrO_2_; *P*(*c*) < 0.05 and *P*(*c* ^*^) < 0.01 compared with Al_2_O_3_; *P*(*d* ^*^) < 0.01 compared with β-TCP.

Figure 1b shows the plates of β-TCP, ZrO_2_, Al_2_O_3_, Ti6Al4V, and PEEK. All the plates had morphological integrity and a sophisticated surface. β-TCP scaffold (Figure 1c) had a complete physical structure and regular micropore morphology. The XRD phase analysis of β-TCP produced by SLA 3DP showed that the distribution of peaks was highly consistent with the characteristics of Ca_3_(PO_4_)_2_ and in agreement with the characteristics of β-TCP of CaP materials (Figure 2). No characteristic peaks of new materials were found. In the Micro-CT reconstruction model (Figure 3a) and the SEM observation (Figure 3b), we can find that the morphology of the β-TCP scaffold is intact and the scaffold has good connectivity and no micropore obstruction. Moreover, we can find that the parameters of the scanning model are consistent with those of the CAD model, which indicates that SLA technology has high accuracy in manufacturing β-TCP scaffold (Table 2).

**Table 2:** Comparison of micropore parameters between Micro-CT and CAD models

	Porosity (%)	Volume (mm^3^)	Specific surface area (mm^−1^)
CAD model	66.13	373.42	7.77
Micro-CT model	64.37	374.12	8.59

### 3.2 Water contact angle

The 0 s water contact angles for bioceramics were relatively small, i.e., 93.32 ± 3.62° and 89.00 ± 6.76° for Al_2_O_3_ and ZrO_2_, respectively. The 0 s water contact angle of β-TCP was the smallest (78.00 ± 2.29°), and significantly lower than PEEK (109.82 ± 13.50°) and Ti6Al4V (106.82 ± 10.40°) (*P* < 0.05) (Figure 3c). After the water droplet contacted the materials, the contact angle reduced due to various causes, such as the water adsorption by porous materials and diffusion of water droplets (Figure 3d). At 1 s after contact, the water contact angle of PEEK (52.02 ± 5.54°) was significantly larger than in other materials, and the water contact angle of Ti6Al4V (38.65 ± 4.17°) was also significantly larger than the other three bioceramics (*P* < 0.05). Among the bioceramics, the water contact angle of β-TCP (9.26 ± 2.57°) was significantly smaller than ZrO_2_ (23.99 ± 4.88°) and Al_2_O_3_ (26.43 ± 4.35°) (*P* < 0.05).

### 3.3 Hemolytic toxicity

**Figure 4:**
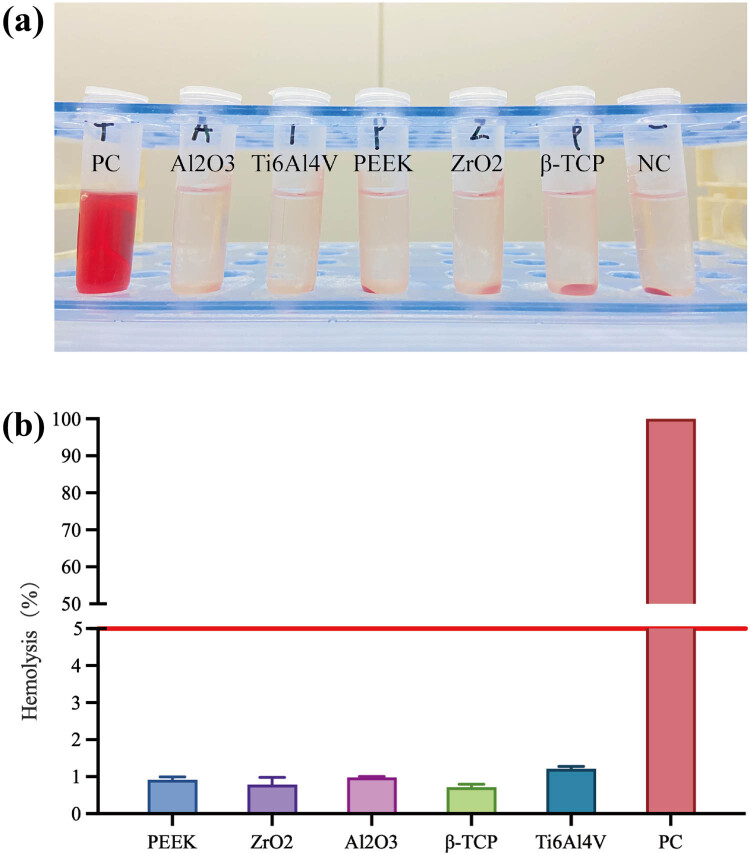
Hemolytic toxicity of different materials. (a) Supernatant of RBCs in the experimental groups, negative control group (NC, with PBS), and positive control group (PC, with distilled water). (b) Hemolysis rates of different materials (<5%) and positive control group (100%).

The supernatant of the negative group and experimental groups showed lucid and transparent supernatant, and no hemolysis was found, while the supernatant in the positive group was bright red and showed evident hemolysis (Figure 4a). The hemolytic toxicity assay (Figure 4b) showed that the hemolysis rates of PEEK, ZrO_2_, Al_2_O_3_, β-TCP, and Ti6Al4V, which were all <5% (0.92 ± 0.08, 0.79 ± 0.19, 0.97 ± 0.03, 0.72 ± 0.08, and 1.21 ± 0.07%, respectively), met the requirement of hemolytic toxicity.

### 3.4 Cell proliferation

**Figure 5:**
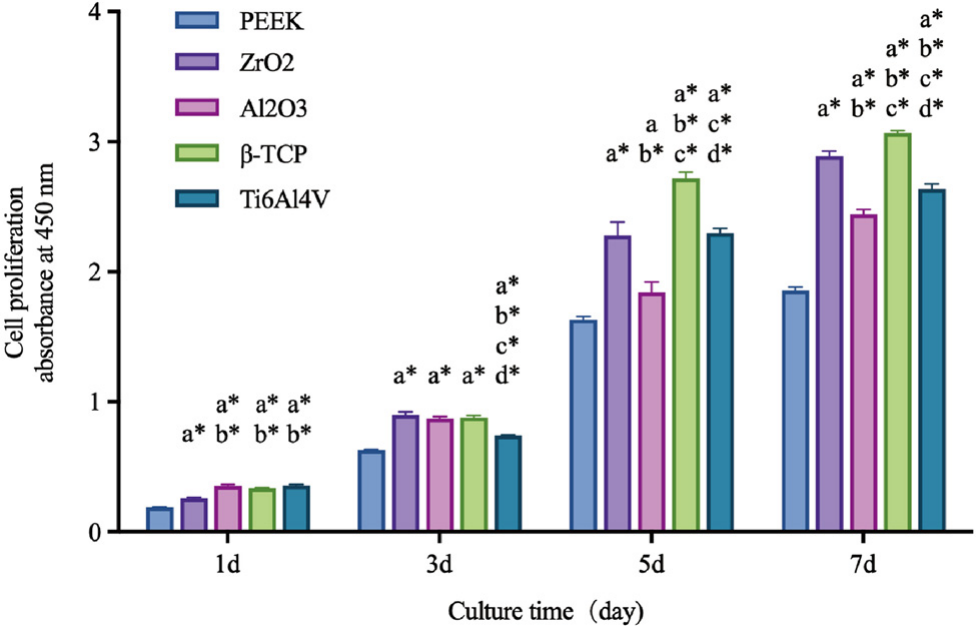
Cell proliferation at different culture times. *P*(*a*) < 0.05 and *P*(*a* ^*^) < 0.01 compared with PEEK; *P*(*b* ^*^) < 0.01 compared with ZrO_2_; *P*(*c* ^*^) < 0.01 compared with Al_2_O_3_; *P*(*d* ^*^) < 0.01 compared with β-TCP.

As shown in Figure 5, the cells on PEEK, ZrO_2_, Al_2_O_3_, β-TCP, and Ti6Al4V all increased with the time of culture. However, the proliferation of cells on PEEK was significantly lower than on the other four materials (*P* < 0.05). Among ceramics, the proliferation of cells on Al_2_O_3_ occurred earlier than on ZrO_2_; however, with the increase of culture time, the cell proliferation on Al_2_O_3_ was lower compared to other ceramics and Ti6Al4V (*P* < 0.05). The proliferation of cells on ZrO_2_ gradually increased on 3 days of culture and was higher than on Ti6Al4V. The proliferation of cells on β-TCP was relatively high and was significantly higher than on other materials on 5 days of culture (*P* < 0.05).

### 3.5 SEM observation

**Figure 6:**
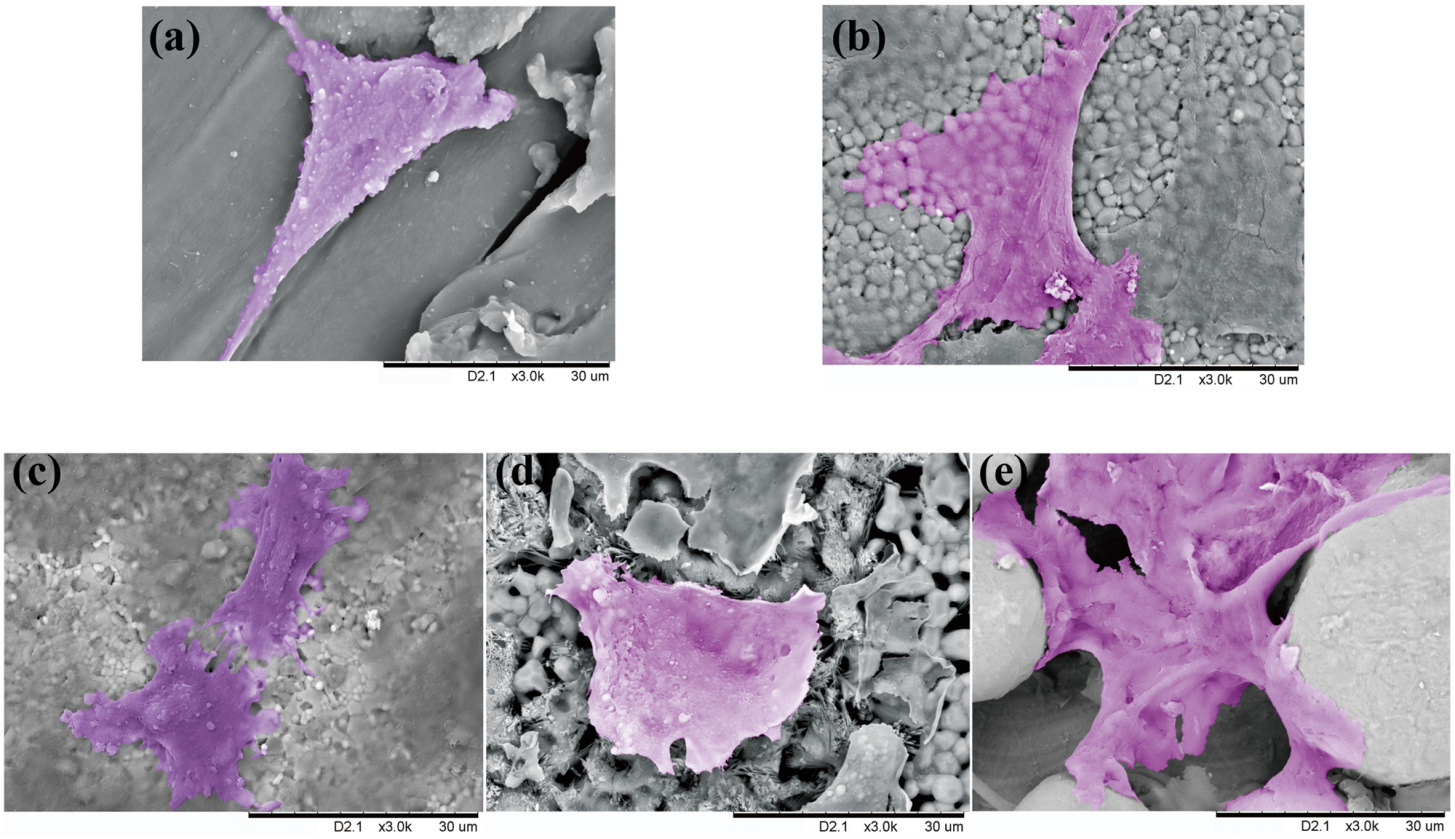
Representative SEM images of the growth of MC3T3 cells on the plates surface. (a) Cells on PEEK had sharp morphologies and fewer pseudopodia. (b–d) Cells with numerous extended pseudopodia were completely expanded and flat on ZrO_2_, Al_2_O_3_, and β-TCP. (e) Cells extend and grow between the sphere particulates on Ti6Al4V.

The low vacuum field emission SEM was used to assess the cell morphology. As shown in Figure 6, cells could adhere to and grow on all plates, but cells that adhered to PEEK had fewer pseudopodia, which had sharp morphologies, while the background was the PEEK material, showing clear texture and smooth surface (Figure 6a). Figure 6b–d shows the ZrO_2_, Al_2_O_3_, and β-TCP. The particulates on the surfaces of the three bioceramics were all evenly distributed, showing porous structures. Cells were completely expanded and flat on the surface of all bioceramics; numerous pseudopodia were extended from the cells, while the bases were wide and with irregular morphologies. Cells on Ti6Al4V material (Figure 6e) showed the adhesive morphologies of the fusion of metal spheres of Ti6Al4V and the ability of cells to grow between the Ti6Al4V sphere particulates. Cells adhered to the adjacent Ti6Al4V sphere particulates and extended numerous processes to adjacent particulates; the bases of cells were wide, and the cells showed plump morphologies.

### 3.6 ALP level

**Figure 7:**
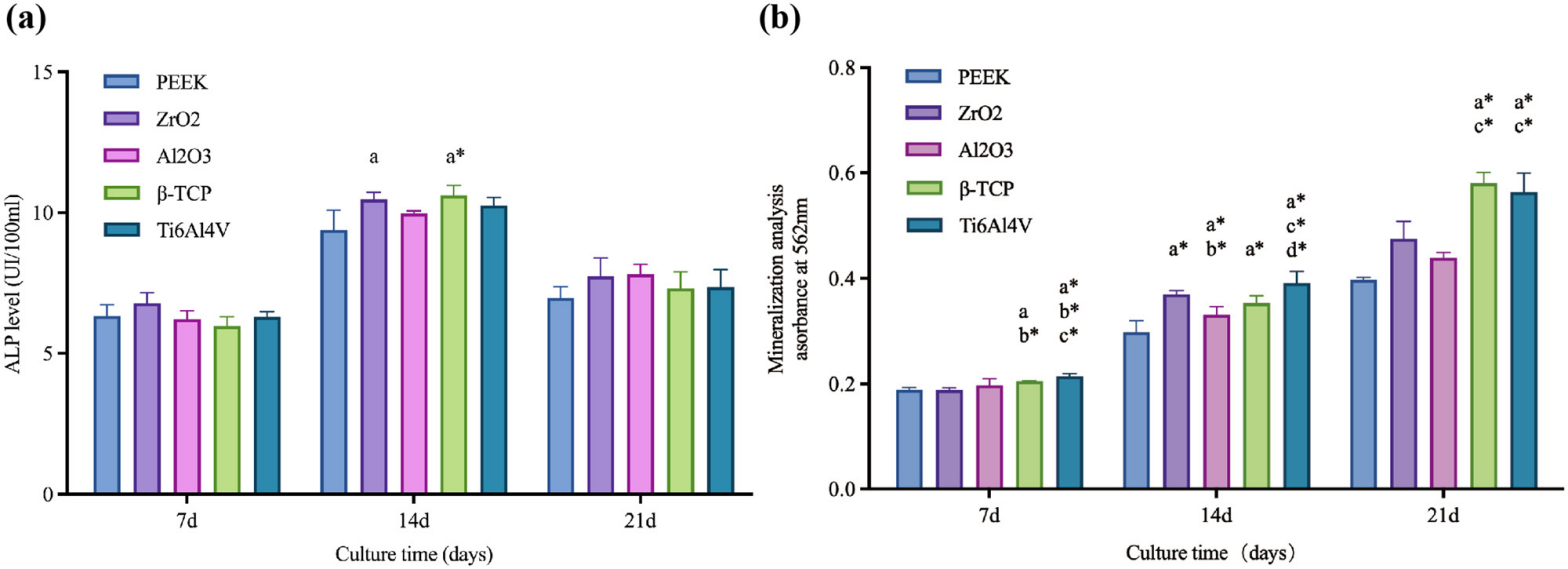
Cell differentiation and osteogenic ability at different culture times. (a) ALP level and (b) the mineralization of extracellular matrix. *P*(*a*) < 0.05 and *P*(*a* ^*^) < 0.01 compared with PEEK; *P*(*b* ^*^) < 0.01 compared with ZrO_2_; *P*(*c* ^*^) < 0.01 compared with Al_2_O_3_; *P*(*d* ^*^) < 0.01 compared with β-TCP.

The synthesis and secretion of ALP by osteoblasts are the markers of early osteogenesis. As shown in Figure 7a, the ALP levels did not significantly differ at the early stage of induction. However, the ALP secretion reached the peak level on 14 days of induction. The ALP level on ZrO_2_ and β-TCP were significantly higher than on PEEK (*P* < 0.05). However, on 21 days of induction, ALP levels of cells on all materials decreased and were not significantly different. The ALP level of cells on β-TCP was not significantly different from Ti6Al4V at any time.

### 3.7 Mineralization

The degree of extracellular matrix mineralization could reflect the maturation of the osteoblast matrix. As shown in Figure 7b, the mineralization was significantly higher on Ti6Al4V than PEEK, ZrO_2_, and Al_2_O_3_, and also significantly higher on β-TCP than PEEK and other ceramics on 7 days of differentiation induction (*P* < 0.05). On 14 days of differentiation induction, the mineralization on PEEK was significantly lower compared to other materials; the mineralization on Al_2_O_3_ was relatively low among ceramics; while the Ti6Al4V had the highest mineralization degree (*P* < 0.05). On 21 days of differentiation induction, the mineralization degree on Ti6Al4V and β-TCP was significantly higher than on other materials (*P* < 0.05).

### 3.8 RT-qPCR

**Figure 8:**
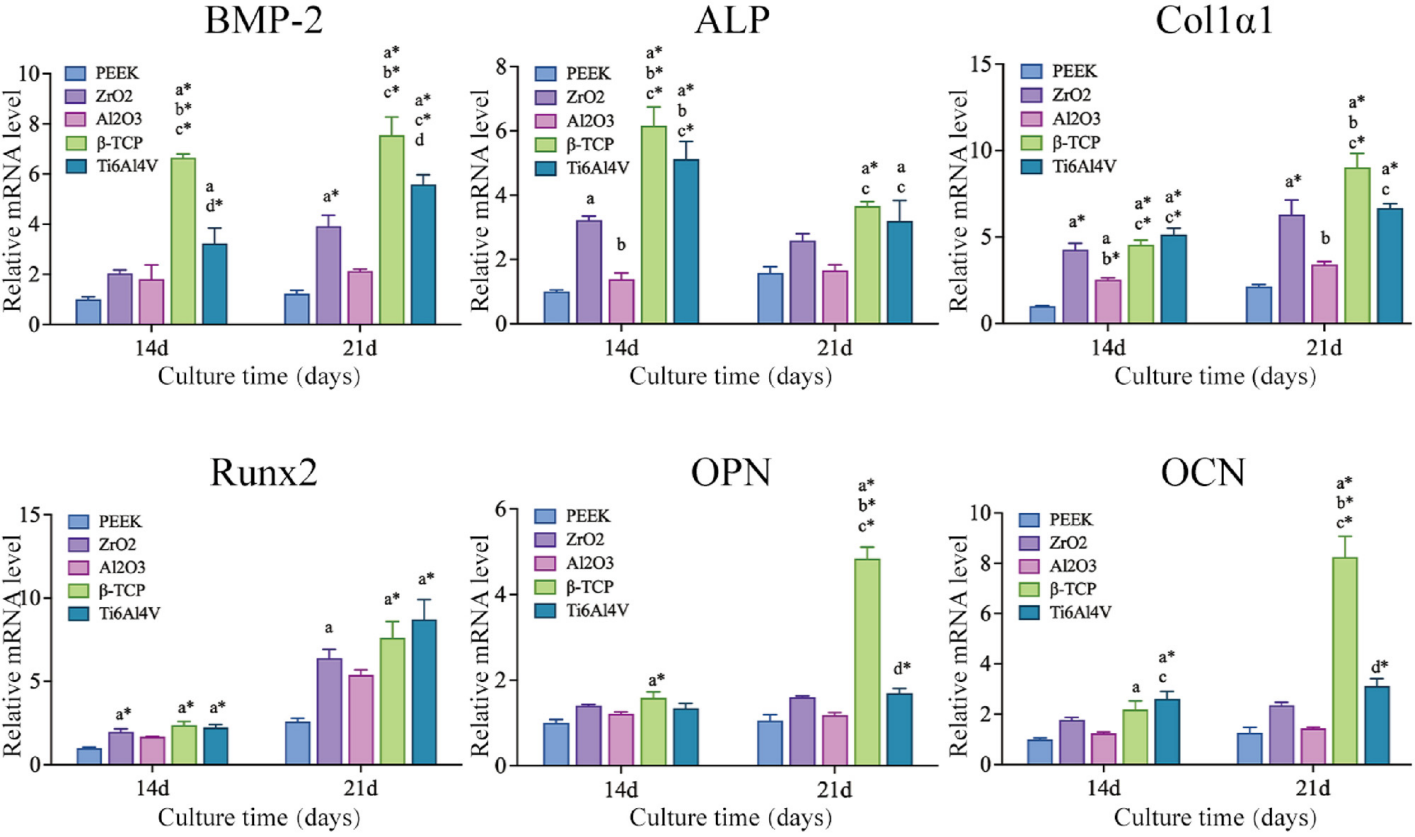
RT-qPCR analysis of osteogenesis-related genes of cells on different material plates. Osteogenesis-related genes including BMP-2, ALP, COL-1, Runx-2, OPN, and OCN. *P*(*a*) < 0.05 and *P*(*a* ^*^) < 0.01 compared with PEEK; *P*(*b*) < 0.05 and *P*(*b* ^*^) < 0.01 compared with ZrO_2_; *P*(*c*) *<* 0.05 and *P*(*c* ^*^) < 0.01 compared with Al_2_O_3_; *P*(*d*) < 0.05 and *P*(*d* ^*^) < 0.01 compared with β-TCP.

Real-time quantitative PCR was used to measure the expression of osteogenesis-related genes after differentiation induction. As shown in Figure 8, the BMP-2 and ALP gene expression on β-TCP and Ti6Al4V were significantly higher compared to other groups and also significantly higher on β-TCP than Ti6Al4V (*P* < 0.05). Compared to PEEK and Al_2_O_3_, cells on ZrO_2_, β-TCP, and Ti6Al4V had significantly higher Col1α1 and Runx2 gene expression (*P* < 0.05). The expression of OPN and OCN genes was significantly higher on β-TCP compared to other materials (*P* < 0.05).

## 4 Discussion

There are various materials for bone implants, among which metal materials have good mechanical performances, but their elastic modulus is relatively high. Polymer materials have similar mechanical performances as human bones; however, their osteogenesis and osteointegration capability are relatively low. Bioceramic has good osteogenesis capability, especially the degradable CaP materials such as β-TCP, which have excellent biocompatibility and osteointegration capability, making them good choices for bone implants [35–37]. Despite the good osteogenesis capability of CaP bioceramics, the micropore structures required for osteogenesis are difficult to be manufactured for CaP due to the limitations of conventional manufacturing techniques. Following the advancements of additive manufacturing techniques, SLA has become one of the effective methods in manufacturing bioceramics with high viscosity and controllable structures. In this study, β-TCP ceramic powder and photosensitive resin were mixed and then printed by SLA 3DP technique to improve the final structure’s precision and controllability. The sizes of printed plates were in agreement with the designed sizes, which had a smooth surface, no fracture, and no burr or protrusion, indicating that the printing had good overall effects. And β-TCP scaffold printed by SLA 3DP had a complete physical structure, regular micropore morphology, and showed good pore connectivity in Micro-CT scan, which means it could be used for the microporous bone implant.

The chemical structures and surface characteristics of materials substantially impact the biological properties [38]. The phase analysis of the β-TCP produced by SLA demonstrated that the chemical additives introduced by 3DP and temperature change for defatting and sintering did not change the phase structures of β-TCP material.

The water contact angle is one of the indexes reflecting the hydrophilic property of the material surface. When the water contact angle is relatively low, water tends to diffuse and adhere to the material, thus indirectly reflecting that the material could favor the cell adhesion, while a high water contact angle reflects the opposite property of the material [39]. When the water droplet immediately contacts the material surface, it becomes plump and larger than the hemisphere. However, the water droplets on β-TCP were relatively flat and lower than the droplets on PEEK and Ti6Al4V. Observation at 1 s after water droplet contact the surface showed that the water droplets diffused on PEEK and Ti6Al4V and water droplet permeation and adsorption were found on the three bioceramics due to the porous structures, which is why the water droplets showed flat morphologies. PEEK had the largest water contact angle than other materials because of the smooth surface and lack of hydrophilic groups, indicating that PEEK had relatively poor cell affinity. Of the three bioceramics, β-TCP had the smallest water contact angle due to the characteristics of the material, as well as the porous structure, which indicated that the material structures of β-TCP could endow it with better cell affinity, favoring the cell adhesion, growth, and proliferation.

The first and foremost biological characteristic of materials for implants is biocompatibility [40]. The effects of materials on blood should first be verified *in vitro* [41]. Several previous studies have reported the clinical application of β-TCP; still, few studies have reported β-TCP produced by steerable SLA 3DP. Therefore, its hemolytic toxicity should be further investigated. The hemolysis rate was <1.5% for all the materials used in the present study. After co-incubation and centrifugation, the RBC aggregated at the bottom of the tube. Although there were several RBC suspended in the supernatant, no hemolysis occurred. Therefore, the β-TCP produced by SLA had no hemolytic toxicity and met the basic clinical application requirements.

The basic biocompatibility of materials is high cell affinity, which allows the cell to adhere, grow, and proliferate on the surface [42–44]. In this study, cells on all materials showed proliferation, but the morphology and proliferation capability varied. PEEK had lower proliferation capability, and cells did not extend fully on its surface, which could be associated with the fact that there were large amounts of hydrophobic groups on the PEEK surface that made cells difficult to grow and proliferate [45]. Ti6Al4V showed good cell proliferation capability and had a high capability of promoting cell adhesion and growth. In comparison to the high cell proliferation rate at an early stage of Al_2_O_3_, ZrO_2_ showed less cell proliferation at the early stage of culture; however, it gradually showed higher cell proliferation with the increase of culture time, which was even higher than Ti6Al4V at the late stage. The relatively lower proliferation of ZrO_2_ at the early stage could be explained by the higher smoothness of the surface [46]. Although the two ceramics had relatively good cell proliferation capability, their toughness and wearing resistance were more prominent, which is why they were mainly used in studies of teeth and joints. β-TCP bioceramic had better cell proliferation capability than Ti6Al4V, which could be attributed to the high similarities of chemical components with human bones, and thus could favor the adhesion and proliferation of osteogenic precursor cells [47]. SEM showed that the surface of β-TCP was unsmooth and had porous structures caused by defatting and sintering. Such structures endow certain water adsorption capability of the material and thus can promote cell adhesion, proliferation, and growth [48].

In addition to the high biocompatibility, bone implant materials also need to have the ability to promote bone formation. Bone-derived ALP mainly binds to the cellular membrane and is closely associated with bone formation, promotion of cell maturation, and mineralization and is the marker of transformation from preosteoblasts to mature osteoblasts. Elevation of ALP activity indicates the initiation of early differentiation. Osteoblasts can absorb the ions and induce mineralization to form calcium nodules. Due to the optical opacity of materials used in this study, quantitative measurements were used to assess the maturity of cell differentiation and mineralization. In the early differentiation induction phase, some preosteoblasts gradually matured, while some other preosteoblasts did not fully differentiate to mature osteoblasts, thus resulting in a lack of significant difference among ALP levels in different groups. At this time, the mature osteoblasts already could integrate the intra- and extracellular ions to induce extracellular matrix mineralization. Therefore, on 7 days of differentiation induction, some mineralized calcium nodules were already detected on the surfaces of Ti6Al4V and β-TCP, indicating that Ti6Al4V and β-TCP could promote extracellular matrix maturation in the early stage. The ALP level peaked with the increase of induction time, and the preosteoblasts generally completed the differentiation to mature osteoblasts. At 2 weeks of induction, β-TCP bioceramic already showed higher osteogenesis capability than PEEK, while the capability of promoting ALP expression in cells was comparable with Ti6Al4V. With the increase of mature osteoblasts, the extracellular matrix gradually matured, and the degree of mineralization also increased. However, PEEK showed lower mineralization compared to other materials. Of the three ceramics, Al_2_O_3_ had the lowest mineralization degree. Although β-TCP had the highest mineralization degree in ceramic materials, it was still lower than Ti6Al4V. Afterward, with the increase of induction time, ALP level gradually reduced, and extracellular matrix mineralization substantially accumulated. After 3 weeks of induction, the degree of extracellular matrix mineralization was still higher on Ti6Al4V and β-TCP than other materials. β-TCP had substantially higher osteogenesis capability at the early stage of osteogenesis than other ceramics and PEEK, and similar excellent performance as Ti6Al4V. In addition, β-TCP had the fastest mineralization speed, suggesting that with the increase of differentiation induction time, β-TCP could have the earliest bone mass formation.

Osteogenesis-related genes are expressed differently to exert the osteogenesis and regulation effects. Such genes include BMP-2, ALP, OPN, OCN, COL-1, and RUNX-2 genes. BMP-2 and analogues can bind to HA and promote the formation of a mineralization core [49]. ALP can degrade inorganic pyrophosphate (PPi) and increase the concentration of inorganic phosphate (Pi), thus promoting the formation of HA crystals and regulating the expression of Runx2 to promote osteogenesis. Previous studies have demonstrated that the ALP gene showed high expression in osteoblasts and vesicles [50]. RUNX-2 is a transcription factor expressed in the early stage of osteogenesis, which could affect cell nuclei to promote the expression of osteogenesis-related genes, such as OPN and OCN, and consequently promote the differentiation of preosteoblasts to osteoblasts [51]. OCN is the major component of non-collagen and the specific marker of bone that is secreted by osteoblasts and promotes calcification. COL1 is the major collagen in the process of osteogenesis that can promote calcification [52]. In this study, the expression of ALP, Col1α1, and Runx2 genes was the early signal of osteogenesis, which indicated the maturation of preosteoblasts to osteoblasts. Such genes had similar expression levels on β-TCP bioceramic and Ti6Al4V. In addition, cells growing on β-TCP bioceramic showed significantly higher levels of BMP-2, OPN, and OCN genes than other materials at different induction phases. With reference to the mineralization, the findings further indicated that β-TCP bioceramic had more powerful capability of promoting the maturation and mineralization of the extracellular matrix of osteoblasts.

In this study, β-TCP produced by SLA has excellent effect in promoting osteogenesis, so it can be used as one of the choice of orthopedic implant in the future. At the same time, SLA method can realize the accurate preparation of high viscosity bioceramics. From this point of view, combined with the fact that the main inorganic components of bone are CaP, this is the key to further promote the application of bioceramics in bone plants, because biodegradable bioceramics scaffolds with fine porous structures are a good choice for artificial bones. Therefore, the results of this study provide a direction for the selection of bone implant materials.

This study has a few limitations. First, due to the optical opacity of materials, some cell markers that should be observed by staining were not involved or were tested and explained by quantitative methods. In addition, more *in vivo* experiments are needed to fully prove the biocompatibility and osteogenic ability of these materials. Finally, our next step is to use SLA technology to process β-TCP bone scaffolds, and study the effects of microporous parameters and morphology of bone scaffolds on osteogenesis.

## 5 Conclusions

During the process of manufacturing and post-processing of SLA 3DP of degradable β-TCP bioceramic in the present study, no evident impurity substance was introduced. In addition, β-TCP plate and scaffold produced by SLA had good integrity and high hydrophilic property. β-TCP produced by SLA had no evident hemolytic toxicity but powerful cell proliferation capability. Cells on β-TCP bioceramic could express higher osteogenesis-related ALP levels, and the mineralization of the extracellular matrix occurred earlier on β-TCP and the maturity was higher. In addition, the expression of osteogenesis-related genes was also very high on β-TCP. These findings demonstrate that the degradable β-TCP bioceramic produced by SLA 3DP has high biocompatibility and osteogenesis capability and could be used as a choice for bone implants in future clinical practice.
